# Implementation and Evaluation of a Text Message–Based Addiction Counseling Program (Text4Hope-Addiction Support): Protocol for a Questionnaire Study

**DOI:** 10.2196/22047

**Published:** 2020-11-17

**Authors:** Vincent Israel Opoku Agyapong, Marianne Hrabok, Wesley Vuong, April Gusnowski, Reham Shalaby, Shireen Surood, Andrew J Greenshaw, Avininder Aulakh, Adam Abba-Aji, Mohit Singh

**Affiliations:** 1 Division of Community Psychiatry Department of Psychiatry, Faculty of Medicine and Dentistry University of Alberta Edmonton, AB Canada; 2 University of Calgary Calgary, AB Canada; 3 Alberta Health Services Edmonton, AB Canada

**Keywords:** addiction, support drugs, alcohol, Text4Hope, mobile phones, text messaging, anxiety, depression, mHealth, COVID-19

## Abstract

**Background:**

With the emergence of the COVID-19 pandemic, providing counseling to people with drug or alcohol addiction while maintaining physical distance has been challenging. This protocol describes the use of text messaging (as used in the Text4Hope-Addiction Support program) as a convenient, evidence-based, cost-effective, and accessible population-level mental health intervention with high user satisfaction proven in prior research.

**Objective:**

The project goal is to implement a program of daily supportive text messaging (Text4Hope-Addiction Support) to reduce drug or alcohol cravings as well as anxiety and depression, typically associated with alcohol and substance use disorders. The aim of this study is to evaluate the prevalence of cravings, anxiety, and depressive symptoms; demographic correlates of the same; and the outcomes of the Text4Hope-Addiction Support intervention in mitigating cravings, anxiety, and depressive symptoms.

**Methods:**

Self-administered, anonymous, online questionnaires will be used to assess cravings for the primary substance of addiction (Brief Substance Craving Scale), anxiety (Generalized Anxiety Disorder-7), and depressive symptoms (Patient Health Questionnaire-9). Data will be collected at baseline (onset of receiving text messages), program midpoint (6 weeks), and program end (12 weeks).

**Results:**

As of October 2020, data collection is in progress; and it is expected to be completed by fall 2021. Data analysis will include parametric and nonparametric techniques, focusing on primary outcomes (ie, cravings, anxiety, and depressive symptoms) and metrics of use, including the number of subscribers and user satisfaction.

**Conclusions:**

This Text4Hope-Addiction Support project will provide key information regarding the prevalence rates of cravings, anxiety, and depressive symptoms among persons with alcohol and substance use disorders; demographic correlates of cravings, anxiety, and depression; and outcome data related to this scalable population-level intervention. Information from this study will be valuable for addiction care practitioners; it will inform the policy and decision making regarding population-level addiction treatment and support during emergencies.

**International Registered Report Identifier (IRRID):**

DERR1-10.2196/22047

## Introduction

### Background

Substance addiction can cause significant impairment in a person's physical, social, and psycho-occupational level of functioning. The lifetime prevalence of substance use was estimated to be 9.9% [1]. Substance abuse and mental illness have globally affected nearly 1 billion people in 2016 [2]; and unfortunately, only 1 in 6 people receives the necessary help toward addressing substance abuse [3]. Alcohol can create a higher level of disability; and sadly, only 24.1% of those with alcohol use disorder ever receive treatment [4]. Not surprisingly, the total number of worldwide deaths due to alcohol misuse alone is higher than the combined number of deaths from tuberculosis, HIV/AIDS, and diabetes [3]. In Canada, substance abuse accounts for about 21% of all deaths and 23% of potential years lost [5].

A systematic review of the 2010 Global Burden of Disease study reported that mental disorders and substance use disorders were 2 leading causes of years lived with disability [6]. The economic cost of substance abuse in Canada was estimated at Can $40 billion (US $30.4 billion) in 2002, with a per capita cost of Can $1267 (US $964) [7].

Substance abuse is common and frequently co-occurs with mood and anxiety disorders. A systematic review and meta-analysis [8] showed a very strong association of alcohol abuse with major depressive disorder (MDD) (OR 2.42, 95% CI 2.22-2.64) and with any anxiety disorder (OR 2.11, 95% CI 2.03-2.19). Similarly, the associations of abuse of illicit substances with MDD and any anxiety disorder were OR 3.8 (95% CI 3.02-4.78) and OR 2.91 (95% CI 2.58-3.28), respectively [8]. The strength of these associations may suggest that the levels of anxiety and depression are potential biomarkers in people with substance use disorder [9,10].

The relationship between substance abuse and mental illness has been argued to account for a significant number of emergency department (ED) visits (mean 5.2), when compared to substance use disorder alone (mean 2.5) [11]. Similarly, substance abuse and comorbid mood disorder, especially MDD, are associated with a high risk of suicide [12,13] and increased health care service utilization [14], which may partly explain the high number of ED visits.

Adequate support for persons with addiction is likely to reduce their ED presentation and can potentially optimize the acute bed utilization needed to support the COVID-19 bed surge and beyond.

There are multifactorial risk factors that can lead to the development of substance abuse. This can include genetic, social, and psychological factors. Familial studies have reported an increased risk of addiction in persons with a genetic predisposition [15,16], but this increased vulnerability is subject to the interplay between gene and environment. Sociodemographic factors such as peer pressure, unemployment, and geographical location can influence the onset and outcome of substance abuse [17]. Psychological factors predicting a person’s vulnerability to substance abuse include a history of trauma, high psychopathology of anxiety, and depression [18,19].

It is important to understand how natural disasters, such as the COVID-19 pandemic, influence the onset of substance abuse and mental illness. This study will examine the association between COVID-19, addiction, anxiety, and depression. A recent study reported that about 53.8% of the sampled population reported a moderate-to-severe level of anxiety during the COVID-19 pandemic [20].

During the COVID-19 pandemic, centers that adopted strategic services to meet the demand of bed surge capacity for COVID-19 cases have seen a reduction in the rate of psychiatric admissions and a decrease in mental health–related ED visits [21]. However, this is likely to precipitate relapse and possibly increases the risk of self-medication with alcohol or other illicit drugs.

There are different approaches to the treatment of substance abuse. Unfortunately, the treatment of comorbid substance abuse and mood disorder remains a challenge for clinicians. The Canadian Network for Mood and Anxiety Treatments (CANMAT) task force recommendations for the management of patients with mood disorders and comorbid substance use disorders reported that there was no significant level 1 evidence on treatment of comorbid mood disorder and substance abuse [22]. A meta-analysis compared the outcomes of contingency management (CM), cognitive behavioral therapy (CBT), and relapse prevention for substance use disorder with about 50.7 % of persons meeting the criteria for alcohol dependence. The result reported a better short-term benefit in the intervention group than in 67% of participants in the control group. This is statistically significant, as the aggregate effect size of active treatment was high (*d*=1.02). The result also showed a disappointing long-term outcome with high dropout rates in CBT (35.3%) and CM (29.4%). The relapse rate showed only 31% abstinence in the treatment group and 13% abstinence in the control group [23]. Another network meta-analysis reported that the combination of CM and community reinforcement was acceptable and effective in patients with a better short- and long-term abstinence, especially in persons with stimulant use disorder [24]. The result showed that the combination of CM plus CBT and CM alone were superior to the treatment-as-usual approach (standardized mean difference [SMD] 0.75, 95% CI 0.31-1.19 and SMD 0.62, 95% CI 0.43-0.80, respectively). Similarly, the combination of CM plus CBT and CM alone had the longest duration of abstinence compared to the treatment-as-usual approach (SMD 0.74, 95% CI 0.43-1.06 and SMD 0.60, 95% CI 0.43-0.76, respectively).

A systematic review of data between 1999-2015 suggested the evolution of text messages as a useful tool in the management of mental illness and addiction [25]. Recent studies have shown the benefits of using an electronic medium, such as the internet or recorded video, for psychoeducation and prevention of substance abuse [26]. However, persons with substance abuse disorder tend to have low motivation, which can limit the use of video or web-based interventions [27,28]. Mobile phones are increasingly becoming standard tools of communication across all age groups. A survey of 389 people with addiction showed that about 83% of the survey participants owned cellphones [29], and the results of a recent randomized controlled trial showed promising benefits of using supportive text messages in the treatment of MDD with comorbid alcohol abuse. However, the study was limited by its small sample size [30]. Other studies on supportive text messages reported improvement in both abstinence and mental health in about 75%-83% of patients with MDD and comorbid alcohol abuse [31]. Our study was designed to use CBT-based supportive text messages for the treatment of substance use disorder. This daily text messaging program was designed and launched as an addiction counseling modality to complement virtual counseling services during the COVID-19 pandemic to reduce cravings, promote recovery, and observe social distancing.

### Objectives

This protocol describes the implementation of the Text4Hope-Addiction Support program in Canada, a low-cost, evidence-based, supportive text messaging service. The objective of the project is to implement a self-subscribing daily supportive text message program to close the addiction counseling gap and reduce cravings, anxiety, and depression-related drug and alcohol addiction among Canadians during the COVID-19 pandemic. The research questions of this study are as follows:

1. What are the prevalence rates of cravings, anxiety, and depressive symptoms in Canada during the COVID-19 pandemic?
2. What are the demographic correlates of cravings, anxiety, and depressive symptoms?
3. Will the Text4Hope-Addiction Support program help to reduce cravings, anxiety, and depressive symptoms among Canadians experiencing psychological distress during the COVID-19 pandemic?

## Methods

### Recruitment, Evaluation Methodology, and Measurement Plan

In the Text4Hope-Addiction Support program, people self-subscribe to receive daily supportive text messages for 6 months by texting the word “Open2Change” to 393939. The program has been the subject of wide publicity, including social media campaigns run by the local provincial mental health foundation and Alberta Health Services, the single provincial government health care provider. Recruitment posters are also used to advertise the program in addiction treatment facilities across Alberta.

The messages built into the program are aligned with an addiction counseling framework, with content written by a clinical psychologist and psychiatrist. This content was then reviewed and revised by a multidisciplinary team consisting of addiction counselors, psychiatrists, and mental health therapists. Some examples of the messages are as follows:

1. In early recovery, your feelings may be more noticeable without masking by substance use. Use more self-care strategies and social support to get you through.
2. Challenge your thoughts about urges. Urges are uncomfortable, but are not unbearable. Urges are distracting, but will not drive you crazy.
3. Addiction is often cue-based. Identify people, places, and things that trigger the desire to use.

The messages are preprogrammed into an online software that delivers messages daily at 8 AM. At the onset of the first message, respondents are welcomed to the service and are invited to complete an online baseline survey capturing demographic information (age, gender, employment status, relationship status, ethnicity, and educational level); COVID-19–related self-isolation/quarantine information; and responses on the Brief Substance Craving Scale (BSCS) [32], Generalized Anxiety Disorder-7 (GAD-7) scale [23], and the Patient Health Questionnaire-9 (PHQ-9) [25]. Survey questions are programmed into REDCap, an online survey tool operated by the University of Alberta. No incentives are offered to respondents. Participation in the program is entirely voluntary. Noncompletion of the survey does not impact the delivery of supportive text messages; this is clearly stated to subscribers in the text messaging program information provided at subscription. Subscribers can opt out of the program at any time by texting “Open2Change” to 393939. Participant consent is indicated via submission of subscribers’ survey responses. Survey responses will be stored within the University of Alberta REDCap account, and data will be exported and analyzed by the Text4Hope research team. Ethics approval has been granted by the University of Alberta Health Research Ethics Board (Pro00086163).

### Sample Size Considerations

The Canadian Community Health Survey 2018 [33] reports that 20% of Canadians experience an alcohol or substance use disorder. Alberta’s population is approximately 4.4 million; and we project that 2.5% of those with alcohol and substance abuse disorders would actively seek help for their addition during the COVID-19 pandemic. Accordingly, we expect about 22,000 Canadians to subscribe to the Text4Hope-Addiction Support program within 12 months of its launch. Based on the response rate of 21.7% for our prior Text4Mood survey [34], we anticipate around 4400 responses to the Text4Hope-Addiction Support surveys in 1 year.

### Outcome Measures

Primary outcomes are changed scores at 6 and 12 weeks from baseline on the BSCS, GAD-7, and PHQ-9 scales. Secondary outcomes are (1) interactions between demographic characteristics of subscribers and primary outcomes and (2) subscriber satisfaction/experience.

### Proposed Timeline and Milestones

The first stage of the study involved the creation and review of supportive text messages, targeting cravings, anxiety, and depression and programming of those messages into the software. This stage was completed on April 20, 2020. The second stage involved the launch of the Text4Hope-Addiction Support program, which occurred on May 22, 2020. The remainder of the project will be focused on data collection, analysis, and reporting.

### Hypotheses

This study will test the following hypotheses:

1. High rates of craving, anxiety, and depression will be reported at baseline, affecting more than half of the sample population.
2. Specific risk demographics and COVID-19–related factors related to the experience of cravings, anxiety, and depression during the pandemic will be identified.
3. The intervention will result in a 25% or greater reduction in cravings, anxiety, and depressive symptoms (as measured by the BSCS, GAD-7, and PHQ-9 scales) at 6 and 12 weeks from baseline. This hypothesis is based on the results of a randomized controlled trial of daily supportive text messaging, which showed that there was close to 25% additional improvement in the Beck Depression Inventory scale–measured mood in the intervention group compared to the control group [30].
4. At least 80% of subscribers will express satisfaction with the Text4Hope-Addiction Support program and perceive the daily supportive text messages as contributing to their overall mental well-being.

## Results

We expect the data collection to be completed by October 2021 and study results to be available in spring 2022. We would implement and evaluate the Text4Hope-Addiction Support program with the Reach, Effectiveness, Adoption, Implementation, and Maintenance (RE-AIM) Framework [26] and the Alberta Quality Matrix for Health [27]. We would include the following dimensions in the evaluation: acceptability (subscriber satisfaction/experience); accessibility (ease of subscription to and utilization of the Text4Hope-Addiction Support program); appropriateness (numbers of persons with drug and alcohol problems subscribing to the program); and effectiveness (6- and 12-week changes in the BSCS, GAD-7, and PHQ-9 scales). It may also be possible to examine efficiency (cost avoidance and efficiencies through reduced need for face-to-face counseling) and safety (self-reports of decreased crisis and urgent service calls and decreased emergency medical services utilization rates).

We will evaluate the efficacy of Text4Hope-Addiction Support with the reductions of cravings, anxiety, and depression at 6 weeks and 12 weeks. Data analysis will include standard use of parametric and nonparametric techniques (eg, within-subject general linear models), including multiple comparison type 1 error corrections. A power analysis with effect sizes from previous studies by Agyapong and colleagues [30,31,34] indicates sufficient effect size for expected Text4Hope-Addiction Support program subscribers’ sample size.

## Discussion

The impacts of COVID-19 on health, lifestyle, psychological safety, and well-being are difficult to overstate. The psychological impact is likely to be more significant for those with mental health conditions and substance use disorders. Mental health support for groups vulnerable to mental health risks during infectious outbreaks requires innovative techniques that can serve the high number of people requiring support while respecting the need to maintain physical distance.

This protocol describes the use of mobile health (mHealth) technology as a convenient, cost-effective, and accessible means for implementing a psychological intervention for people with substance use disorders during the pandemic. This program is empirically supported in previous research, which show good outcomes as well as high user satisfaction.

This study will evaluate outcomes according to the standardized and empirically validated questionnaires. It will provide key information regarding the prevalence rates of substance use cravings, anxiety, and depression during the COVID-19 pandemic; demographic correlates; and outcome data (cravings, anxiety, and depression). Information from this study will thus be critical for practitioners as well as useful for informing policy and decision making regarding psychological interventions during the COVID-19 pandemic, especially for persons with mental health and substance use disorders. We hope that the outcome of this study will inform the integration of supportive text messaging into treatment programs readily available to persons with substance use disorders.

## Abbreviations

BSCS: Brief Substance Craving Scale
CANMAT: Canadian Network for Mood and Anxiety Treatments
CBT: cognitive behavioral therapy
CM: contingency management
ED: emergency department
GAD-7: Generalized Anxiety Disorder-7
MDD: major depressive disorder
mHealth: mobile health
PHQ-9: Patient Health Questionnaire-9
RE-AIM: Reach, Effectiveness, Adoption, Implementation, and Maintenance
SMD: standardized mean difference

## References

[1] Grant BF, Saha TD, Ruan WJ, Goldstein RB, Chou SP, Jung J, Zhang H, Smith SM, Pickering RP, Huang B, Hasin DS (2016). Epidemiology of DSM-5 Drug Use Disorder: Results From the National Epidemiologic Survey on Alcohol and Related Conditions-III. JAMA Psychiatry.

[2] Rehm J, Shield KD (2019). Global Burden of Disease and the Impact of Mental and Addictive Disorders. Curr Psychiatry Rep.

[3] (2019). Global Status Report on Alcohol and Health 2018. World Health Organization.

[4] Hasin DS, Stinson FS, Ogburn E, Grant BF (2007). Prevalence, correlates, disability, and comorbidity of DSM-IV alcohol abuse and dependence in the United States: results from the National Epidemiologic Survey on Alcohol and Related Conditions. Arch Gen Psychiatry.

[5] Single E, Robson L, Rehm J, Xie X, Xi X (1999). Morbidity and mortality attributable to alcohol, tobacco, and illicit drug use in Canada. Am J Public Health.

[6] Whiteford HA, Degenhardt L, Rehm J, Baxter AJ, Ferrari AJ, Erskine HE, Charlson FJ, Norman RE, Flaxman AD, Johns N, Burstein R, Murray CJ, Vos T (2013). Global burden of disease attributable to mental and substance use disorders: findings from the Global Burden of Disease Study 2010. Lancet.

[7] Rehm J, Gnam W, Popova S, Baliunas D, Brochu S, Fischer B, Patra J, Sarnocinska-Hart A, Taylor B (2007). The costs of alcohol, illegal drugs, and tobacco in Canada, 2002. J Stud Alcohol Drugs.

[8] Lai HMX, Cleary M, Sitharthan T, Hunt GE (2015). Prevalence of comorbid substance use, anxiety and mood disorders in epidemiological surveys, 1990-2014: A systematic review and meta-analysis. Drug Alcohol Depend.

[9] Kessler RC, Crum R M, Warner L A, Nelson C B, Schulenberg J, Anthony J C (1997). Lifetime co-occurrence of DSM-III-R alcohol abuse and dependence with other psychiatric disorders in the National Comorbidity Survey. Arch Gen Psychiatry.

[10] Willinger Ulrike, Lenzinger Elisabeth, Hornik Kurt, Fischer Gabriele, Schönbeck Georg, Aschauer Harald N, Meszaros Kurt, European fluvoxamine in alcoholism study group (2002). Anxiety as a predictor of relapse in detoxified alcohol-dependent patients. Alcohol Alcohol.

[11] Curran GM, Sullivan G, Williams K, Han X, Allee E, Kotrla KJ (2008). The association of psychiatric comorbidity and use of the emergency department among persons with substance use disorders: an observational cohort study. BMC Emerg Med.

[12] Oquendo MA, Currier D, Liu S, Hasin DS, Grant BF, Blanco C (2010). Increased risk for suicidal behavior in comorbid bipolar disorder and alcohol use disorders: results from the National Epidemiologic Survey on Alcohol and Related Conditions (NESARC). J Clin Psychiatry.

[13] Blanco C, Alegría Analucía A, Liu S, Secades-Villa R, Sugaya L, Davies C, Nunes EV (2012). Differences among major depressive disorder with and without co-occurring substance use disorders and substance-induced depressive disorder: results from the National Epidemiologic Survey on Alcohol and Related Conditions. J Clin Psychiatry.

[14] Cassidy F, Ahearn EP, Carroll BJ (2001). Substance abuse in bipolar disorder. Bipolar Disord.

[15] Hancock DB, Markunas CA, Bierut LJ, Johnson EO (2018). Human Genetics of Addiction: New Insights and Future Directions. Curr Psychiatry Rep.

[16] Hall FS, Drgonova J, Jain S, Uhl GR (2013). Implications of genome wide association studies for addiction: are our a priori assumptions all wrong?. Pharmacol Ther.

[17] Kidd JD, Tross S, Pavlicova M, Hu M, Campbell ANC, Nunes EV (2017). Sociodemographic and Substance Use Disorder Determinants of HIV Sexual Risk Behavior in Men and Women in Outpatient Drug Treatment in the NIDA National Drug Abuse Treatment Clinical Trials Network. Subst Use Misuse.

[18] Regier DA, Farmer M E, Rae D S, Locke B Z, Keith S J, Judd L L, Goodwin F K (1990). Comorbidity of mental disorders with alcohol and other drug abuse. Results from the Epidemiologic Catchment Area (ECA) Study. JAMA.

[19] Ai AL, Lee J (2018). Childhood Abuse, Religious Involvement, and Lifetime Substance Use Disorders among Latinas Nationwide. Subst Use Misuse.

[20] Wang C, Pan R, Wan X, Tan Y, Xu L, Ho CS, Ho RC (2020). Immediate Psychological Responses and Associated Factors during the Initial Stage of the 2019 Coronavirus Disease (COVID-19) Epidemic among the General Population in China. Int J Environ Res Public Health.

[21] Li W, Yang Y, Liu Z, Zhao Y, Zhang Q, Zhang L, Cheung T, Xiang Y (2020). Progression of Mental Health Services during the COVID-19 Outbreak in China. Int J Biol Sci.

[22] Beaulieu S, Saury S, Sareen J, Tremblay J, Schütz Christian G, McIntyre RS, Schaffer A, Canadian Network for MoodAnxiety Treatments (CANMAT) Task Force (2012). The Canadian Network for Mood and Anxiety Treatments (CANMAT) task force recommendations for the management of patients with mood disorders and comorbid substance use disorders. Ann Clin Psychiatry.

[23] Dutra L, Stathopoulou G, Basden SL, Leyro TM, Powers MB, Otto MW (2008). A meta-analytic review of psychosocial interventions for substance use disorders. Am J Psychiatry.

[24] De Crescenzo F, Ciabattini M, D'Alò Gian Loreto, De Giorgi R, Del Giovane C, Cassar C, Janiri L, Clark N, Ostacher MJ, Cipriani A (2018). Comparative efficacy and acceptability of psychosocial interventions for individuals with cocaine and amphetamine addiction: A systematic review and network meta-analysis. PLoS Med.

[25] Watson T, Simpson S, Hughes C (2016). Text messaging interventions for individuals with mental health disorders including substance use: A systematic review. Psychiatry Res.

[26] Hopson L, Wodarski J, Tang N (2015). The effectiveness of electronic approaches to substance abuse prevention for adolescents. J Evid Inf Soc Work.

[27] McKellar J, Kelly J, Harris A, Moos R (2006). Pretreatment and during treatment risk factors for dropout among patients with substance use disorders. Addict Behav.

[28] Ilgen MA, McKellar J, Moos R, Finney JW (2006). Therapeutic alliance and the relationship between motivation and treatment outcomes in patients with alcohol use disorder. J Subst Abuse Treat.

[29] Milward J, Day E, Wadsworth E, Strang J, Lynskey M (2015). Mobile phone ownership, usage and readiness to use by patients in drug treatment. Drug Alcohol Depend.

[30] Agyapong VI, Ahern S, McLoughlin DM, Farren CK (2012). Supportive text messaging for depression and comorbid alcohol use disorder: single-blind randomised trial. J Affect Disord.

[31] Agyapong VIO, Milnes J, McLoughlin DM, Farren CK (2013). Perception of patients with alcohol use disorder and comorbid depression about the usefulness of supportive text messages. Technol Health Care.

[32] Alcohol and Drug Abuse Institute Library (1995). Brief Substance Craving Scale (BSCS).

[33] (2019). Canadian Community Health Survey, 2018. Statistics Canada.

[34] Agyapong Vio, Mrklas K, Juhás Michal, Omeje J, Ohinmaa A, Dursun Sm, Greenshaw Aj (2016). Cross-sectional survey evaluating Text4Mood: mobile health program to reduce psychological treatment gap in mental healthcare in Alberta through daily supportive text messages. BMC Psychiatry.

